# University Public-Access Mandates Are Good for Science

**DOI:** 10.1371/journal.pbio.1000237

**Published:** 2009-11-10

**Authors:** David Shulenburger

**Affiliations:** Association of Public and Land-Grant Universities, Washington, D.C., United States of America

## Abstract

A new and better order to make scholarship available for free to all is emerging through deposit mandates like those adopted by Harvard, MIT, and Kansas.

“*The faculty of Arts and Sciences of Harvard University is committed to disseminating the fruits of its research and scholarship as widely as possible*.”

Why would university faculty choose to place their scholarship on electronic archives for a world-wide audience? Many US universities have adopted such mandates for public access to faculty research, perhaps most notably Harvard [1], MIT, and the University of Kansas [2]. These policies (and many more like them in various stages of consideration on campuses across the nation and world) are harbingers of a new order, one in which essentially all scholarly articles can be found and accessed by any interested individual.

This spring, the Association of Public and Land-Grant Universities, the Association of American Universities, the Association of Research Libraries, and the Coalition for Networked Information sent a document entitled “The Research University's Role in the Dissemination of Research and Scholarship,” [3] to all public and private US research universities, requesting that serious campus discussion on the topic occur. The document resulted from a roundtable of officers of the four associations and 21 provosts, research officers and librarians, and university press representatives, invited from their member universities. There is much to be gained by enlarging the universe of those who have full access to scholarship. Ubiquitous campus public-access deposit mandates will rapidly generate this gain.

## Ending the Age of Disorder

The last 25 years have been an age of disorder, not an unusual beginning for a revolution. Stewart Brand's declaration at the dawn of the digital age that “information wants to be free” foretold the porous electronic world that scholarship has come to inhabit. In the 25 years since Brand uttered those words, scholarly works have grown increasingly free. That which, prior to the digital age, could be found only within the covers of the scholarly journal, first emerged from those covers as electronic replacements for working papers. Unlike the mimeographed and later photocopied versions of papers, the new electronic versions could be circulated without cost and, even after hundreds of reproductions, remain readable.

Soon, the informal digital circulation of working papers was followed by Web posting. Those far beyond the author's mailing list could get copies of the work. The first stirrings of the arXiv occurred in August 1991 and rapidly grew as a means of facilitating sharing of physics article preprints and post-prints. Other disciplines—funding agencies, national libraries, and universities—copied this innovation. The Directory of Open Access Repositories [4] now reflects the existence of 1,440 repositories world-wide, with roughly 80% housed in institutions, 13% hosted by disciplines, and the rest government- or aggregator-focused.

A diligent electronic search for most any article or manuscript today will produce the item itself or some version of it. However, what one finds often will reflect the disorderly nature of this age. Unfortunately, many of the hits will be accessible only if one has a subscription to the journal, is part of an institutional community that has a subscription, or is willing and able to pay for the manuscript on an ad hoc basis. Many researchers find that these hurdles inhibit their research. Surveying 2,157 US scientists in 2007, Stephen Hansen of the American Association for the Advancement of Science found that 29% of respondents said that their own research had been affected by difficulties in gaining access to or disseminating copyrighted scientific literature [5].

“Difficulties with obtaining access to or disseminating scientific literature” may mean that specific articles could not be found, that a version “of record” could not be found, or that multiple versions of an article were found, leaving the researcher unable to determine which version properly might be cited. Sources that are not curated and/or associated with stable URLs can be found one day and then vanish the next.

And the opportunity cost of blocking access to potentially valuable information increases as understanding of science grows. Those who already suffer from what Robert Merton dubbed “the Matthew effect” [6], in which eminent scientists receive greater recognition for their work than do unknown researchers, are placed at a further disadvantage by the exponential increase in scientific publications. Researchers must deal with the near impossibility of keeping up with “the flood of published science research, even in one's own narrow field.” For example, Thinh Nguyen of the Science Commons reported (Universal Access Digital Library Summit, Boston, MA, September 25, 2008) that 128,000 papers have been written on apoptosis arising from the genes and proteins that may be associated with Huntington's disease and the similarly vast numbers of papers on the gene and cell interactions that may be implicated in autism. This “vastly increased bulk of publication stiffens the competition,” Merton wrote—made all the worse by anything that makes papers harder to read.

While serving as head of the National Institutes of Health (NIH), Elias Zerhouni observed that “we have no one place where the integration of the information can be used as a powerful hypothesis generator” [7]. He set about to produce the desired order by continuing the work begun by his predecessor at NIH, Harold Varmus, building PubMed Central as a partial solution for the biomedical sciences. It has become a large, though not complete, corpus of the biosciences/biomedical literature. It will be more complete in the future because articles arising from NIH grants accepted for publication after April 7, 2008, must be deposited in PubMed Central.

## The Emerging New Order

The only solution that gives science the maximum chance for advancement is one that ensures that all science findings are available to all researchers. “Available” does not permit permanent subscription or price barriers to stand between the researcher and scientific findings. When potentially important works that may bear on one's research number in the tens of thousands, “available” means that crawlers with sophisticated artificial intelligence must also have full access to help sort through the mass.

Public access mandates from funding agencies and foundations like NIH and the Wellcome Trust are part of the solution, but not all of it. While deposit mandates should be universally adopted by funders, such agencies support only a fraction of the work that is published in scholarly journals. Large portions of important work in most fields originate beyond US borders. Most work outside the physical and biological sciences is not funded by grants external to the university and will not be touched by such mandates. Given that important problems are seldom bounded by a single discipline's research, access to the non-science scholarly literature is potentially important to all researchers.

The most effective method of ensuring that the majority of important work is available is by replicating across the academy university public-access mandates like those of Harvard, MIT, and Kansas throughout the world. Most works originate with university-affiliated faculty or have co-authors who are faculty members. Deposit of articles in the form in which they were published in a journal requires permission of journals that require that authors provide exclusive copyright to them. In the Harvard policy, the faculty member grants a “nonexclusive, irrevocable, paid up, world-wide license to exercise any and all rights under copyright” to Harvard College [8]. While these provisions can be waived by the Dean in exceptional circumstances, the language sends a strong message to the journal that if it wishes to publish papers of the Harvard faculty, it will not object to inclusion of the articles in Harvard's repository. The MIT and Kansas policies have like provisions. When complemented by funding agency and foundation public-access mandates that capture the work originating with industry and government researchers who may not have faculty status, university mandates will, in time, produce nearly universal access to all the scientific literature.

## Public Access for the Intermediate Term

Note that I use the term “public access” rather than “open access.” Fortunately, open-access journals like those of BMC and PLoS have found a way to make open access work. Unfortunately, most of the scholarly literature journals depend on the subscription model and feel threatened by immediate open access to the material they publish. While open access is the desired goal in the long term, the same logic that compelled PubMed Central to design itself as “public access”—with up to a year's embargo permitted to protect the subscription base of journals—compels me to support public access as an interim measure. Public access permits the possibility of brief embargoes at the request of the journal of publication, in contrast to open access, which requires that access to full text and databases, without permission restrictions, occur immediately.

Journals opposing open access often claim that it will take away the funding needed for the refereeing process. Clearly the refereeing process must be supported. I know of no rigorous evidence that even very brief embargo periods before making articles publicly available cause scientific journal subscriptions to decline; therefore, I believe that public access has little impact on subscription revenue and is thus fully consistent with ensuring that refereeing of the literature continues.

An explicit tradeoff between having access to all scholarly journal articles after no more than one year's delay is preferable to running even a small risk that immediate access would damage the refereeing process. In the long run, it will be incumbent on any journal insisting that access be delayed to produce evidence that the harm done to science by delayed access is less than the harm that would be done to science if immediate access were provided. As more and more scholarly journals change their practices and permit immediate posting on publicly accessible Web sites, it will be increasingly difficult to defend the position that short embargo periods cause harm to journals.

## Is This an Expensive Solution?

In this period of great financial stress for universities, the question of the cost of maintaining public-access repositories must be addressed. Fortunately, most US research universities already have operating repositories in which public-access–mandated collections may be placed. For the few institutions that do not, repository software is available for free [9] or organizations like the Berkeley Electronic Press will provide, for a very modest annual fee, a turn-key solution for establishing a repository that includes both the needed software and mass storage.

The future of all libraries is digital. Most collection access is now through electronic means. To argue that maintaining a digital archive of faculty scholarly articles will be too expensive is essentially to argue that the university will be unable to maintain a viable library resource in the future.

## Benefits to Universities

Not many taxpayers know what university faculty are doing. In fact, not many university administrators or even other faculty know what research their colleagues are performing. This veil over faculty research may contribute to the 20-year trend of declining real per-student subsidy from states to their institutions of higher education. The decline in real state support is especially pronounced at research universities.

University public-deposit mandates will enhance the ability of universities to demonstrate faculty research productivity to the citizens of their states and to their donors. Imagine the massive collection of research that universities will accumulate after five years of mandated deposits. Further imagine alerting the public and donor community to the ability to search university X's repository to discover what local faculty findings exist on any subject. The results of such a search—on subjects ranging from stem cells to menopause and hair loss—would be impressive. Suddenly the invisible campus becomes a place populated by individuals researching topics relevant to the average citizen. Legislators who complain about faculty productivity would find their arguments more difficult to sustain. Donors and potential donors might even alter their gift-giving based on such searches.

## Your Opportunity and Responsibility

As a careful observer of scholarly communications, I'm convinced that the public goods aspect of faculty research will ultimately compel public access to it. Public goods have the characteristic that use of them by one individual does not diminish their value to others. In fact, the knowledge presented through scholarship generally becomes more valuable as it is shared more widely and becomes a building block upon which further scientific advances may occur.

Faculty members can accelerate the process. We can persuade colleagues on our own campuses to pass public-access mandates like those at Harvard, MIT, and Kansas. We can speed up what otherwise might be a 20-year process and make it happen in three or four. We can urge Congress to expand the NIH mandate to all federal funding agencies [10]. We can convince the less-enlightened scholarly societies that representing our disciplines means working for public access to scholarship rather than opposing it.

It is impossible to know how much more rapidly scientific progress will occur if all the scholarly literature becomes accessible. What we each know is the frustrations we've experienced in our own research because of access difficulties. It is within the power of the university faculty in this country to remove these roadblocks. Supporting adoption of a public-access deposit mandate on your campus is an effort most worthy of the involvement of dedicated scientists.
